# Enthusiasm for cancer screening in Great Britain: a general population survey

**DOI:** 10.1038/bjc.2014.643

**Published:** 2014-12-23

**Authors:** J Waller, K Osborne, J Wardle

**Affiliations:** 1 Health Behaviour Research Centre, Department of Epidemiology and Public Health, University College London, Gower Street, London WC1E 6BT, UK; 2 Cancer Research UK, Angel Building, 407 St John Street, London EC1V 4AD, UK

**Keywords:** screening attitudes, cancer screening, beliefs about screening, mass screening/psychology

## Abstract

**Background:**

With growing concerns about risk of harm from cancer screening, particularly from overdiagnosis, this study aimed to assess public attitudes to cancer screening in Great Britain.

**Methods:**

We used a population-based survey to assess attitudes to cancer screening, screening history and demographic characteristics, in men and women aged 50–80 years. Data were collected using face-to-face computer-assisted interviews in 2012.

**Results:**

In our sample of 2024, attitudes to cancer screening were overwhelmingly positive with almost 90% believing that screening is ‘almost always a good idea' and 49% saying they would be tested for cancer even if it was untreatable. Attitudes were particularly positive among those who had previously taken part in breast or colorectal screening.

**Conclusions:**

Our findings suggest that attitudes to cancer screening are very positive in Great Britain. Widespread enthusiasm for cancer screening may hamper attempts to encourage a greater appreciation of the limitations and potential harms of screening.

Provision of information about cancer screening has been the subject of much debate in recent years, particularly in relation to mammography where it has been argued that the risks are downplayed (Jørgensen and Gøtzsche, 2006; Gøtzsche *et al*, 2009). In the United Kingdom, information materials for all three national cancer screening programmes (breast, cervical and colorectal) have recently been revised, with the aim of providing balanced information to allow people to make an informed choice about participation (Richards, 2011). The underlying model is ‘consider an offer' (Entwistle *et al*, 2008), with a clear National Health Service (NHS) recommendation, but also direct advice to make an individual decision (Informed Choice about Cancer Screening, 2014). However, people's screening decisions are also likely to be influenced by prevailing public attitudes and, in some cases, their own past behaviour.

A population survey carried out in the United States in 2001–2002 found very high levels of enthusiasm for cancer screening (Schwartz *et al*, 2004). They speculated that this could be due to pervasive marketing of screening and a public discourse in which the limitations of screening are rarely discussed. However, it could also be a consequence of the intuitive appeal of ‘catching cancer early'. Although such enthusiasm may promote screening uptake, it can also lead to dissatisfaction when guidelines are revised to reduce the recommended frequency or age range for screening (Squiers *et al*, 2011; Arkes and Gaissmaier, 2012). It may also hamper efforts to implement an informed choice approach because potential participants see no reason to revisit an ‘obvious' choice.

Cancer screening in Great Britain takes place in a different context from the United States. Direct-to-consumer marketing is uncommon, there are few national public health campaigns on screening, and primary-care physicians have little involvement in programme delivery (particularly for colorectal screening). Screening is offered using organised call-recall programmes, in which routine invitations are sent to all age-eligible adults alongside information on risks and benefits. The public in Great Britain has also recently been exposed to media debate about the risks and benefits of breast screening, including considerable publicity surrounding a review of the mammography programme (Richards, 2011). We assessed enthusiasm for cancer screening in this context; hypothesising that attitudes in the Great Britain might be less positive than in the United States 10 years earlier.

## Methods

We commissioned a survey on attitudes to cancer screening in spring 2012 as part of TNS International's regular omnibus survey (TNS International is the name of the research agency we commissioned to carry out the fieldwork for this study). This is a weekly, population-based, face-to-face survey, in which clients can commission questions. Sampling points across England, Scotland and Wales are selected using random location sampling, stratified by region and social grade. Local quotas are used to balance the sample of adults interviewed at eligible addresses and to adjust for likelihood of being at home. Quotas are set for gender (male/female housewife/female non-housewife), work status and presence of children. The survey uses home-based, computer-assisted, personal interviews.

### Participants

Adults aged 50–80 years were eligible. All women in this age-group would have been offered breast screening. Since 1988, women aged 50–70 years have been invited for mammography every 3 years, and the programme is currently being extended to ages 47–49 and 71–73. Both men and women would have been around the age of eligibility for colorectal screening. People aged 60–69 years have been offered biennial screening with faecal occult blood testing since 2006 (with slight variations in age-ranges between the England, Scotland and Wales), and once-only flexible sigmoidoscopy screening is currently being implemented at age 55 years (since 2013).

### Measures

We included items from the earlier US survey (Schwartz *et al*, 2004) to assess enthusiasm for screening (see Table 1 for item wording). We also asked participants whether they had ever been screened for breast or colorectal cancer and collected demographic information.

### Analyses

Analyses were carried out using PASW Statistics 18.0 (SPSS Inc., 2009). Weights provided by TNS were applied to the data in all analyses to adjust the sample to be population-representative with respect to age, gender, social class and geographical region. Logistic regression analyses were used to calculate adjusted odds ratios for endorsement of each screening attitude by demographic characteristic and screening participation.

## Results

### Sample characteristics

The overall weighted sample size was 1895 (51%, *n*=975 women). Mean age was 63.0 (s.d.=8.6), most participants were married (60%), and white (94%), and 39% had no educational qualifications (Appendix 1). Among those who were age-eligible for screening at the time of the survey, 62% had already had colorectal cancer (CRC) screening (58% of men and 66% of women) and 86% of women had attended breast screening.

### Overall beliefs

Enthusiasm for screening was extremely high (Table 1). Nearly 90% of respondents agreed that screening is ‘almost always a good idea', 75% believed that earlier detection means treatment can save lives ‘most' or ‘all' the time, and 64% thought early diagnosis means less treatment is needed ‘most' or ‘all' the time. Around half (49%) were previously unaware that some cancers are slow-growing and unlikely to cause problems, but 45% of the total sample wanted to be tested for such a cancer. A similar proportion (49%) wanted to be tested for a cancer for which nothing could be done. Almost 60% regarded declining a screening offer as ‘irresponsible', and 72% believed they had received the right number of screening tests in the past.

### Demographic variations in beliefs

In adjusted analyses (see Table 2), the oldest age group were less likely to believe that finding cancer early means that treatment saves lives (odds ratio (OR)=0.67, 95% confidence interval (CI): 0.51–0.89) and was less likely than the younger age group to believe they had too few screening tests in the past (see Table 2). Men were more likely than women to want to be tested for an incurable cancer (OR=1.32, 95% CI: 1.08–1.61) or for a slow-growing cancer (OR=1.29, 95% CI: 1.06–1.57) and were also more likely to think they had received too few screening tests in the past (OR=2.82, 95% CI: 2.26–3.54). Men were less likely to have heard of a slow-growing cancer (OR=0.72, 95% CI: 0.59–0.88). Participants with no educational qualifications were more likely to think that screening non-attendance is irresponsible (OR=1.26, 95% CI: 1.02–1.56) but less likely to have heard of a slow-growing cancer (OR=0.47, 95% CI: 0.38–0.57). Those of non-white ethnicity were less likely to believe screening is a good idea (OR= 0.27, 95% CI: 0.15–0.50) and that screening non-attendance is irresponsible (OR=0.56, 95% CI: 0.34–0.91). Those who were not married were less likely to be aware of a slow-growing cancer (OR=0.77, 95% CI: 0.63–0.94).

### Associations between beliefs and previous screening attendance

After controlling for demographic variables, non-participation in CRC screening was associated with less positive beliefs about the benefits of screening, lower awareness of slow-growing cancers, less enthusiasm for being tested for untreatable or slow-growing cancers, lower odds of believing that non-attendance is irresponsible and higher odds of believing that one has had too few screening tests in the past (see Table 2).

A similar pattern of findings was observed for breast screening, but there was no association between previous attendance and either awareness of, or desire to be tested for, a slow-growing cancer. The association with the belief that finding cancer early means less treatment was also non-significant.

## Discussion

Our findings suggest that enthusiasm for cancer screening in Great Britain in 2012 is at least as high as in the United States 10 years earlier (Schwartz *et al*, 2004). The association between past screening attendance and enthusiasm was not universal and was, to some extent, programme-specific. It is striking that enthusiasm was so high despite screening participation (in this sample and in the wider UK population) being considerably lower –54% for CRC screening nationally (von Wagner *et al*, 2011). This is consistent with the idea that, although people might ‘know' that screening is a good idea, they may hold contradictory beliefs or not get round to participating.

Men's greater enthusiasm for being tested for slow-growing and incurable cancers may be the result of lower exposure to screening and less familiarity with issues of overdiagnosis. This, together with their belief that they have had too few screening tests, is consistent with positive attitudes to prostate specific antigen (PSA) testing in men (Chapple *et al*, 2008). This is a key area for future investigation.

The similarity in attitudes in Great Britain and the United States raises the possibility that positive appraisal of screening is less due to marketing and public health campaigns (as Schwartz *et al* (2004) suggest), because both are less pervasive in Britain, but arises from a lay logic that earlier interception of the oncogenic process is beneficial, perhaps reinforced by public discourse around the value of prevention and early diagnosis. In addition, the fact that many respondents in both countries would want to know about an untreatable cancer suggests that knowledge of health status *per se* is valued. Taken together, these results highlight the challenge of communicating the benefit/harm balance of any particular screening modality (see for example, Brawley *et al*, 2011) and suggest that the public is probably relatively unresponsive to media debate on overdiagnosis.

The study has a number of limitations, the most important of which is that TNS International are not able to supply information on response rate. Although weighting the data goes some way to adjusting for non-response bias, the generalisability of the findings is uncertain given the unknown response rate.

Medical concerns about an ‘epidemic' of overdiagnosis (Hoffman and Cooper, 2012) will be hard to communicate in the presence of such enthusiasm. Positive attitudes towards screening may make participation a reflexive process, inhibiting a ‘rational' appraisal of risk/benefit information. These findings indicate the need for further work to explore ways of encouraging active engagement with screening decisions.

## Figures and Tables

**Table 1:** Enthusiasm for screening

**Item**	**Response**	***N*** **(%)**
Do you think routine cancer screening tests for	Yes	1682 (88.8)
healthy people are almost always a good idea?	No	137 (7.3)
	Do not know	75 (4.0)
How often does finding cancer early mean that treatment saves lives?	None	19 (1.0)
	Some	462 (24.4)
	Most	982 (51.8)
	All the time	432 (22.8)
How often does finding cancer early mean that	None	50 (2.6)
a person can have less treatment?	Some	630 (33.2)
	Most	915 (48.3)
	All the time	301 (15.9)
Have you ever heard of cancers that grow so	Yes	873 (46.1)
slowly that they are unlikely to cause you any	No	929 (49.0)
problems in your lifetime?	Do not know	92 (4.9)
Would you want to be tested to see if you had a	Yes	861 (45.4)
slow-growing cancer like that?	No	876 (46.2)
	Do not know	158 (8.3)
If there was a kind of cancer for which nothing	Yes	931 (49.1)
could be done, would you want to be tested to	No	774 (40.8)
see if you had it?	Do not know	190 (10.1)
In the past, do you think you have had too many	Too few	504 (26.6)
screening tests for cancer, too few or about the	About right	1360 (71.8)
right number	Too many	31 (1.6)
Do you feel that someone [who] does not go for	Yes	1112 (58.7)
screening is irresponsible?	No	624 (32.9)
	Do not know	159 (8.4)

**Table 2:** Associations between screening beliefs, demographic factors and screening experience

	**Routine screening is a good idea**		**Finding cancer early means treatment saves lives**		**Finding cancer early means less treatment**		**Not going for screening is irresponsible**	
	**Yes** ***n*** **(%)**	**Adjusted OR for responding ‘yes' (95% CI)**	**Most/all of the time** ***n*** **(%)**	**Adjusted OR for ‘most/all the time' (95% CI)**	**Most/all of the time** ***n*** **(%)**	**Adjusted OR for ‘most/all the time' (95% CI)**	**Yes** ***n*** **(%)**	**Adjusted OR for ‘yes' (95% CI)**
**Demographic factors**								
Age								
50–59 years	652 (93.0)	1.00	555 (76.7)	1.00	460 (63.5)	1.00	402 (60.5)	1.00
60–69 years	606 (92.9)	0.95 (0.60–1.51)	508 (75.4)	0.84 (0.64–1.10)	438 (65.0)	1.00 (0.79–1.27)	411 (65.7)	1.21 (0.95–1.54)
70–80 years	424 (90.8)	0.68 (0.42–1.08)	351 (70.6)	**0.67** (**0.51–0.89)**	318 (63.9)	0.98 (0.77–1.26)	298 (67.1)	1.21 (0.94–1.56)
Gender								
Women	861 (92.0)	1.00	722 (74.1)	1.00	615 (63.1)	1.00	597 (66.5)	1.00
Men	821 (93.0)	1.13 (0.79–1.61)	692 (75.2)	1.10 (0.86–1.37)	600 (65.3)	1.12 (0.92–1.37)	515 (61.5)	0.82 (0.67–1.01)
Qualifications								
Any	1021 (92.6)	1.00	847 (74.6)	1.00	728 (64.1)	1.00	651 (61.4)	1.00
None	654 (92.4)	0.99 (0.68–1.46)	556 (75.2)	1.16 (0.92–1.45)	477 (64.5)	1.05 (0.86–1.28)	456 (68.1)	**1.26** (**1.02–1.56)**
Ethnicity								
White	1604 (93.2)	1.00	1343 (75.3)	1.00	1157 (64.9)	1.00	1070 (64.9)	1.00
Non-white	72 (80.9)	**0.27** (**0.15–0.50)**	67 (69.1)	0.63 (0.39–1.03)	56 (57.7)	0.71 (0.45–1.12)	38 (48.7)	**0.56** (**0.34–0.91)**
Marital status								
Married	1019 (93.0)	1.00	862 (76.1)	1.00	746 (65.9)	1.00	657 (63.1)	1.00
Not married	663 (91.7)	0.80 (0.56–1.14)	552 (72.4)	0.83 (0.66–1.03)	470 (61.6)	0.82 (0.68–1.00)	454 (65.4)	1.01 (0.82–1.24)
**Screening experience**								
Colorectal^a^								
Ever screened	522 (95.4)	1.00	448 (79.4)	1.00	392 (69.5)	1.00	383 (72.0)	1.00
Never screened	295 (89.4)	**0.39** (**0.24–0.66)**	229 (66.2)	**0.49** (**0.36–0.66)**	195 (56.5)	**0.55** (**90.41–0.73)**	180 (57.9)	**0.50** (**0.37–0.68)**
Breast^a^								
Ever screened	586 (94.5)	1.00	495 (78.0)	1.00	414 (65.2)	1.00	411 (69.8)	1.00
Never screened	82 (81.3)	**0.27** (**0.14–0.52)**	66 (64.7)	**0.52** (**0.33–0.82)**	57 (55.9)	0.71 (0.46–1.10)	47 (49.0)	**0.45** (**0.28–0.71)**

	**Want to be tested for a cancer for which nothing can be done**		**Heard of a slow-growing cancer**		**Want to be tested for a slow-growing cancer**		**Too few screening tests in the past**	
	**Yes** ***n*** **(%)**	**Adjusted OR (95% CI)**	**Yes** ***n*** **(%)**	**Adjusted OR (95% CI)**	**Yes** ***n*** **(%)**	**Adjusted OR (95% CI)**	**Too few** ***n*** **(%)**	**Adjusted OR (95% CI)**
**Demographic factors**								
Age								
50–59 years	369 (56.8)	1.00	336 (48.6)	1.00	340 (50.9)	1.00	252 (34.8)	1.00
60–69 years	335 (54.1)	0.89 (0.70–1.14)	332 (51.4)	1.25 (0.99–1.58)	309 (50.2)	0.94 (0.74–1.19)	155 (23.0)	**0.53** (**0.41–0.69)**
70–80 years	227 (52.1)	0.87 (0.68–1.12)	206 (44.1)	1.07 (0.84–1.38)	211 (46.6)	0.83 (0.65–1.06)	97 (19.5)	**0.46** (**0.35–0.60)**
Gender								
Women	447 (51.2)	1.00	479 (51.2)	1.00	412 (46.2)	1.00	169 (17.3)	1.00
Men	484 (58.2)	**1.32** (**1.08–1.61)**	394 (45.4)	**0.72** (**0.59–0.88)**	449 (53.1)	**1.29** (**1.06–1.57)**	334 (36.3)	**2.82** (**2.26–3.54)**
Qualifications								
Any	588 (56.9)	1.00	609 (55.6)	1.00	522 (49.4)	1.00	330 (29.0)	1.00
None	339 (51.2)	0.84 (0.68–1.03)	261 (37.2)	**0.47** (**0.38–0.57)**	336 (49.9)	1.10 (0.90–1.34)	171 (23.2)	0.91 (0.72–1.14)
Ethnicity								
White	887 (55.0)	1.00	835 (48.8)	1.00	816 (49.5)	1.00	463 (26.0)	1.00
Non-White	39 (48.1)	0.68 (0.42–1.11)	36 (43.4)	0.78 (0.48–1.29)	40 (50.0)	0.96 (0.59–1.56)	36 (37.5)	1.26 (0.76–2.08)
Marital status								
Married	572 (56.5)	1.00	554 (51.3)	1.00	531 (51.0)	1.00	314 (27.7)	1.00
Not married	358 (51.8)	0.89 (0.73–1.09)	319 (44.1)	**0.77** (**0.63–0.94)**	330 (47.3)	0.90 (0.74–1.10)	190 (24.9)	1.08 (0.86–1.35)
**Screening experience**								
Colorectal^a^								
Ever screened	310 (60.2)	1.00	299 (54.7)	1.00	290 (55.9)	1.00	101 (17.9)	1.00
Never screened	140 (44.3)	**0.50** (**0.37–0.66)**	127 (39.4)	**0.60** (**0.45–0.79)**	127 (40.3)	**0.50** (**0.37–0.67)**	98 (28.4)	**1.82** (**1.30–2.55)**
Breast^a^								
Ever screened	315 (54.0)	1.00	325 (52.4)	1.00	290 (49.9)	1.00	103 (16.2)	1.00
Never screened	38 (41.3)	**0.59** (**0.37–0.93)**	53 (55.2)	1.21 (0.78–1.88)	37 (39.4)	0.65 (0.41–1.03)	32 (31.7)	**2.09** (**1.27–3.45)**

All odds ratios are adjusted for age, gender, education, ethnic group and marital status. ‘Do not know' responses are treated as missing in all analyses. Sample sizes vary due to missing data. Bold indicates a significant odds ratio (*P*<0.05)

a Analyses include only age-eligible participants.

**Appendix 1 tbla1:** Sample characteristics

	***N*** **(%) unweighted**	***N*** **(%) With weights applied^a^**
All	2024	1895
**Gender**		
Male	951 (47.0)	920 (48.5)
Female	1073 (53.0)	975 (51.5)
**Age group**		
50–59 years	658 (32.5)	724 (38.2)
60–69 years	723 (35.7)	674 (35.6)
70–80 years	643 (31.8)	498 (26.3)
**Marital status**		
Married	1167 (57.7)	1132 (59.8)
Single/separated/widowed/divorced	857 (42.3)	763 (40.2)
**Ethnicity**		
White	1914 (94.6)	1784 (94.1)
Non-white	96 (4.7)	97 (5.1)
**Educational qualifications**		
None	871 (43.0)	738 (39.0)
Any educational qualifications	1132 (57.0)	1136 (60.6)
**Colorectal screening history (*n*=1028)**		
Eligible and ever attended	636 (61.9)	564 (62.0)
Eligible and never attended	392 (38.1)	345 (38.0)
**Breast screening history (*n*=777)**		
Eligible and ever attended	666 (85.7)	635 (86.2)
Eligible and never attended	111 (14.2)	101 (13.8)

a Weights are used to adjust the sample to be representative of the wider population of Great Britain with respect to gender, age, social class and geographical region. As weights may be <1, the overall sample size is reduced when weights are applied.

## Appendix 1

[Table tbla1]
