# Enthusiasm for cancer screening in Great Britain: a general population survey

**DOI:** 10.1038/bjc.2016.138

**Published:** 2016-05-26

**Authors:** J Waller, K Osborne, J Wardle

**Correction to:** *British Journal of Cancer* (2015) **112**, 562–566; doi:10.1038/bjc.2014.643; published online 23 December 2014

**Updated online 26 May 2016:** This article was originally published under a CC BY-NC-SA 4.0 license, but has now been made available under a CC BY 4.0 license. The PDF and HTML versions of the paper have been modified accordingly.

